# Long term worsening of amyloid pathology, cerebral function, and cognition after a single inoculation of beta-amyloid seeds with Osaka mutation

**DOI:** 10.1186/s40478-023-01559-0

**Published:** 2023-04-22

**Authors:** Marina Célestine, Muriel Jacquier-Sarlin, Eve Borel, Fanny Petit, Jean-Baptiste Perot, Anne-Sophie Hérard, Luc Bousset, Alain Buisson, Marc Dhenain

**Affiliations:** 1 Laboratoire Des Maladies Neurodégénératives, Université Paris-Saclay, CEA, CNRS, 18 Route du Panorama, 92265 Fontenay-Aux-Roses, France; 2 Commissariat À L’Energie Atomique Et Aux Énergies Alternatives (CEA), Direction de La Recherche Fondamentale (DRF), Institut François Jacob, MIRCen, 18 Route du Panorama, 92265 Fontenay-aux-Roses, France; 3 Univ. Grenoble Alpes, Inserm, U1216, Grenoble Institut Neurosciences, GIN, 38000 Grenoble, France

**Keywords:** Alzheimer’s disease, Cerebral connectivity, Amyloid-β, Aβ Osaka, Memory, Synapses

## Abstract

**Abstract:**

Alzheimer’s disease (AD) is characterized by intracerebral deposition of abnormal proteinaceous assemblies made of amyloid-β (Aß) peptides or tau proteins. These peptides and proteins induce synaptic dysfunctions that are strongly correlated with cognitive decline. Intracerebral infusion of well-defined Aβ seeds from non-mutated Aβ_1-40_ or Aβ_1-42_ peptides can increase Aβ depositions several months after the infusion. Familial forms of AD are associated with mutations in the amyloid precursor protein (APP) that induce the production of Aβ peptides with different structures. The Aβ Osaka (Aβ_osa_ mutation (E693Δ)) is located within the Aβ sequence and thus the Aβ_osa_ peptides have different structures and properties as compared to non-mutated Aβ_1-42_ peptides (Aβ_wt_). Here, we wondered if a single exposure to this mutated Aβ can worsen AD pathology as well as downstream events including cognition, cerebral connectivity and synaptic health several months after the inoculation. To answer this question we inoculated Aβ_1-42_-bearing Osaka mutation (Aβ_osa_) in the dentate gyrus of APP_swe_/PS1_dE9_ mice at the age of two months. Their cognition and cerebral connectivity were analyzed at 4 months post-inoculation by behavioral evaluation and functional MRI. Aβ pathology as well as synaptic density were evaluated by histology. The impact of Aβ_osa_ peptides on synaptic health was also measured on primary cortical neurons. Remarkably, the intracerebral administration of Aβ_osa_ induced cognitive and synaptic impairments as well as a reduction of functional connectivity between different brain regions, 4 months post-inoculation. It increased Aβ plaque depositions and increased Aβ oligomers. This is the first study showing that a single, sporadic event as Aβ_osa_ inoculation can worsen the fate of the pathology and clinical outcome several months after the event. It suggests that a single inoculation of Aβ regulates a large cascade of events for a long time.

**Graphical Abstract:**

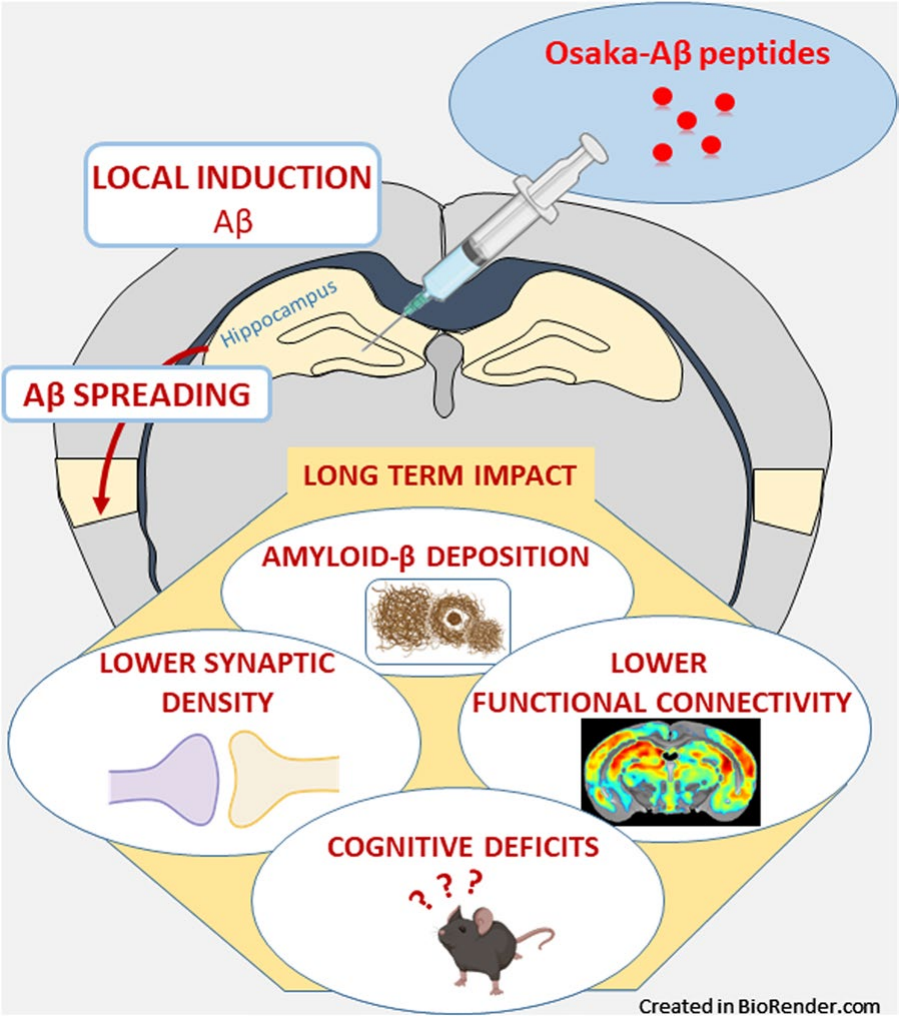

## Introduction

Alzheimer’s disease (AD) is a neurological disorder leading to cognitive deficits. It is characterized by intracerebral accumulation of abnormal proteinaceous assemblies made of amyloid-β (Aß) peptides or tau proteins. These peptides and proteins induce synaptic dysfunctions that are strongly correlated with the cognitive decline [20]. Aβ peptides arise from the proteolytic cleavage of the Aβ precursor protein (APP), leading to monomeric Aβ peptides that can progressively aggregate to form fibrillary deposits (Aβ plaques).

Several studies in humans [6, 14, 17] and in experimental animal models of AD [12, 19, 30, 31] indicate that intracerebral inoculation of minimal amounts of misfolded Aβ present within AD brain extracts induces build-up of Aβ deposits in their host. The intracerebral infusion of well-defined non-mutated Aβ_1-40_ or Aβ_1-42_ peptides can also increase Aβ plaque depositions several months after the infusion [43, 49].

In addition to Aβ pathology, the inoculation of AD-brain extracts can induce long-term cognitive alterations as well as synaptic impairments in rodents [24] or primates [12, 25]. As inoculation of AD-brain extracts can induce both Aβ and tau pathologies [24], it is difficult to dissociate the role of Aβ or tau on cognitive or synaptic impairments induced by this inoculation [24]. Thus, inoculation of synthetic or recombinant Aβ seeds was proposed to evaluate the impact of Aβ on brain function. However, although intracerebral infusion of these Aβ seeds was shown to induce acute memory impairments in wild-type mice [2, 8], to our knowledge, their long term effects have not been reported on cognition or synaptic impairments.

Some familial forms of AD are associated with mutations in the Aβ fragment of the APP. The Osaka mutation (E693Δ, "Osa") is found in a Japanese pedigree of familial AD [46]. It corresponds to amino acid 693 deletion from APP gene, resulting in mutant Aβ peptide (Aβ_osa_) lacking a glutamic amino acid residue at the 22^nd^ position. Thus, as the mutation is located within the Aβ fragment, the Aβ_osa_ peptides have a different structure and properties as compared to Aβ_wt_ [37]. Aβ_osa_ is more resistant than other Aβ species to degradation by major Aβ–degrading enzymes [46]. Synthetic Aβ_osa_ have unique aggregation properties of enhanced oligomerization and no fibrillization [46]. This can be explained by the fact that they exhibit preferred conformational states that allow higher hydrophobicity resulting in faster oligomerization [15]. Consistent with the nonfibrillogenic property of the Aβ_osa_, a very low amyloid plaque load was observed in patient with this mutation [46] and transgenic mice with this mutation APP and they do not show any Aβ plaques even at 24 months but display an age-dependent accumulation of Aβ oligomers within neurons [29, 45]. Given the different structure of Aβ_osa_ peptides and their properties in vitro and in vivo, we wondered if exposure to Aβ_osa_ can worsen AD pathology as well as downstream events including cognition, cerebral connectivity and synaptic health several months after the inoculation. The long-term effect of a single intra-hippocampal inoculation of Aβ_osa_ seeds was evaluated four months post inoculation in transgenic mice (APP_swe_/PS1_dE9_) overexpressing Aβ_1-42_ peptide and presenting Aβ plaques [24]. Exposure to Aβ_osa_ induced cognitive and functional impairments, including reduced hippocampal connectivity compared to mice inoculated with non-mutated Aβ_1-42_ peptide (Aβ_wt_). Exposure to Aβ_osa_ also led to lower synaptic density and increased Aβ plaque load and Aβ oligomers. Our results suggest that the seeding induced by a single exposure to Aβ_osa_ worsen the fate of Aβ pathology and clinical outcome for several months.

## Methods

### Production of recombinant Aβ proteins

To make the plasmids for the fusion protein Aβ(His) of wild-type human β-amyloid 1–42 protein (Aβ_wt_) or Aβ_osa_ mutant (E22Δ), the cDNA containing the sequence for human Aβ_1-42_ was obtained from synthetic oligonucleotides (Sigma, Lyon, France) (containing a Nde1 restriction site as forward primers and a PspXI restriction site as reverse primers) using overlapping PCR. PCR products were then cloned into a pet28a-vector (Novagen, Paris, France) and subsequently constructed as various mutant HIS-Aβ_1-42_ expressing plasmid (pet28a-Aβ(His)_wt_ or pet28a-Aβ(His)_osa_ (E22Δ). The resulting plasmids were verified by sequencing. Escherichia Coli BL21 (DE3) was transformed with the fusion protein plasmids and a single colony was chosen to grow in a 250 mL starter culture in Luria broth (LB medium) overnight at 37 °C. The next day, 10 mL of culture was diluted in 1L LB culture medium. When the culture reached an OD600nm of 0.8, isopropyl-beta-D-thiogalactopyranoside (IPTG) was added to 1 mM for induction. The culture was grown for an additional 4 h and the cells harvested by centrifugation at 4000 g for 20 min. The pellet was re-suspended in 10 mL ice-cold PBS and lysed by sonication at ice-cold temperature. The cell extract was then centrifuged at 20,000 g for 15 min at 4 °C. The pellet was re-suspended in 10 mL of 8 M urea in PBS and sonicated as previously described before centrifugation at 20,000 g for 15 min at 4 °C. The supernatant (5 mL) was diluted with 15 mL of binding buffer (PBS with 10 mM imidazole at pH 8.0). Before affinity purification using nickel-nitriloacetic acid (NTA) column purification, samples were filtered on 0.45 μm. The Ni–NTA column (3 mL of protino Ni–NTA Agarose from Macherey Nagel) was equilibrated with binding buffer prior to loading the sample on the column. Then the column was washed with the washing buffer (PBS with 30 mM imidazole at pH 8.0) with 5–10 column volumes. The protein was then eluted with the elution buffer (PBS with 500 mM imidazole at pH 7.4). The absorbance at 280 nm was used to monitor the elution but the concentration of the fusion proteins was estimated by comparing the intensity of the band of the protein on SDS-PAGE with that of a known quantity of BSA (Sigma, Lyon, France). A final concentration of 100 μM was obtained and aliquots were stored at −80 °C. Aliquots from all subsequent purification steps were analyzed by SDS-PAGE [23], and the identity of Aβ_1-42_ and mutants was verified by western blots using 4G8 monoclonal antibodies against Aβ sequence (4G8).

### Endotoxin assay

Endotoxin content of Aβ solutions was detected using a kinetic Limulus amebocyte lysate (LAL) chromogenic endotoxin quantitation kit (Thermo Scientific). In brief, 50 μM of Aβ solution was prepared in PBS and was transferred to a sterile 96-well plate prewarmed to 37 °C. LAL (0.1 mL, room temperature) was quickly added to each well. Detection relied on standards supplied in the kit with the range from 0.10 to 1 EU/ml and on positive and negative controls that were performed at the same time as the samples. Endotoxin concentrations were determined by measuring kinetic absorbance at 405 nm at 37 °C following the instructions of the manufacturer, in a Spark plate reader (Tecan).

### Thioflavin T (ThT) binding assay

The aggregative properties of different forms of Aβ can be evaluated in vitro with the well-characterized thioflavin dye binding assay [27]. First, the lyophilized synthetic Aβ42 was purchased from Covalb (Villeurbanne, France). It was dissolved at a concentration of 1 mM in DMSO. Aβ monomers stock solutions were generated by dilution of the peptides to a concentration of 100 µM in phosphate buffer, pH 7.4.

Spontaneous fibril formation was evaluated by incubating 8 µM of Aβ monomer solutions at 37 °C for 24 h in presence of 10 μM ThT (Wako Chemical Industries Ltd, Osaka, Japan) in phosphate buffer, pH 7.4. Fibril formation was followed by monitoring ThT fluorescence with shaking using a Hitachi F-2500 fluorometer. The excitation and emission wavelengths were 445 nm and 485 nm, respectively. Fluorescence was determined by averaging the three readings and subtracting the ThT blank.

To perform the seeding experiments, Aβ_wt_ and Aβ_osa_ solutions were rapidly thawed out and were added to the synthetic Aβ_1-42_ + ThT solution previously described. All seeding experiments were performed with 10% or 2% of freshly prepared seeds and 90% or 98% of soluble Aβ monomers, resulting in a total of 8 µM. Aβ_wt_ was used at 10% and Aβ_osa_ at 2 and 10%.

### Electron microscopy

Aβ solutions (100 µl sample of 8 µM) and fibrils obtained from 24 h ThT binding assay were concentrated tenfold by centrifugation at 50,000 g for 10 min and suspended in MilliQ water. Assemblies were layered on glow discharged carbon coated 400 mesh copper grid, and stained with 1% uranyl acetate. The assemblies were observed under Jeol 1400 electron microscope at 80 kV and 10 K magnification. Images were recorded on Rios CCD camera (Gatan).

### Primary cultures of cortical neurons

Mouse cortical neurons were cultured from 14- to 15-day-old OF1 embryos (Charles River) as described previously [26]. After extraction of the embryonic brains, the cerebral membranes were removed and the cortices were dissected, mechanically dissociated and cultured in Dulbecco’s Modified Eagle’s Medium supplemented with 5% horse serum, 5% fetal bovine serum and 1 mM glutamine (all from Sigma) on 24-well plates (Falcon; Becton Dickinson) for biochemical experiments.

Neurons were seeded on 35 mm glass-bottom dishes (MatTek) at a final concentration of two cortical hemispheres per dish for confocal experiments. All plates, dishes, and coverslips were coated with 0.1 mg/mL poly-D-lysine and 0.02 mg/mL laminin (Sigma). Cultures were maintained at 37 °C in a humidified atmosphere containing 5% CO2/95% air. After 3–4 days in vitro (DIV), cytosine arabinoside (AraC, 10 µM; Sigma) was added to inhibit proliferation of non-neuronal cells in cultures used for biochemistry experiments; 98% of the cells were considered as neuronal. The day before the experiments, cells were washed in DMEM. Treatments were performed on neuronal cultures at 14–15 DIV.

### Quantification and morphological characterisation of dendritic spine density

Neurons were visualized using a Nikon Ti C2 confocal microscope with a Nikon 60X water-immersion objective and NIS-Elements software (Nikon, Melville, NY, USA). Excitation of GFP and mCherry fluorophores was performed with an argon laser at 488 nm (emission filtered at 504–541 nm) and at 543 nm (emission filtered at 585–610 nm) respectively. Images (1024 × 1024 pixels) were acquired as Z-stacks (tridimensional section) with 0.3 μM per step. The acquired images were then deconvoluted using AutoQuantX3 software (Media Cybernetics, Abingdon, Oxon, UK). For analysis of spines, serial image files corresponding to z-stacks of 20–30 optical sections per dendritic segment were directly processed with NeuronStudio, a software package specifically designed for spine detection and analysis (https://biii.eu/neuronstudio, CNIC – Mount Sinai School of Medicine). Voxel size was 0.3 x 0.3 x 0.3 µm. After modeling of the dendrite surface, protrusions with a minimum volume of 5 voxels, length of between 0.2 μm and 4 μm and a maximal width of 3 μm were retained as spines. Following default settings of the program and the empirical classification rule previously described [35], spines with a minimum head diameter of 0.35 μm and minimum head vs neck ratio of 1.1 were classified as mushroom spines. Non-mushroom spines with minimum volume of 10 voxels (0.040 μm^3^) were classified as stubby spines. All other spines were considered thin.

### Transgenic mice

Mouse experiments involved the APP_swe_/PS1_dE9_ mouse model of amyloidosis (C57Bl6 background) [11]. Aβ plaques can be detected as early as 4 months of age in these mice and increase in number and total area with age [11]. At the time of the inoculation of Aβ_wt_, Aβ_osa_ or PBS, at 2 months of age, these mice did not have Aβ plaques. Animals were studied for four months after intracerebral inoculation (at 4 post-inoculation (mpi) respectively, *n*_*APP/PS1-Aβwt*_ = 10, *n*_*APP/PS1-Aβosa*_ = 10, *n*_*APP/PS1-PBS*_ = 10). Wild-type littermates injected with the PBS were used as controls for the behavioral tests (*n*_*WT-PBS*_ = 10). All APP_swe_/PS1_dE9_ and littermate WT mice were born and bred in our center (Commissariat à l’Energie Atomique, Fontenay-aux-Roses; European Institutions Agreement #B92-032–02). Females were exclusively used in this study in order to optimize group homogeneity (Aβ plaque load is known to vary between males and females). Mice were injected during different inoculation sessions and each group was randomly inoculated at each session to avoid an "order of treatment" confounding effect. All animals were randomly assigned to the experimental groups using a simple procedure: They were identified using increasing numbers based on their birthdate. Animals with increasing numbers were alternatively assigned to the *APP/PS1-*Aβ_*wt*_ (animal 1, 4, 7…), *APP/PS1-*Aβ_*osa*_ (animal 2, 5, 8…) and *APP/PS1-*_*PBS*_ groups (animal 3, 6, 9…). All experimental procedures were conducted in accordance with the European Community Council Directive 2010/63/UE and approved by local ethics committees (CEtEA-CEA DSV IdF N°44, France) and the French Ministry of Education and Research, and in compliance with the 3R guidelines. Animal care was supervised by a dedicated in-house veterinarian and animal technicians. Human endpoints concerning untreatable continuous suffering signs and prostrations were taken into account and not reached during the study. Animals were housed under standard environmental conditions (12-h light–dark cycle, temperature: 22 ± 1 °C and humidity: 50%) with ad libitum access to food and water. The design and reporting of animal experiments were based on the ARRIVE reporting guidelines [5]. Sample size was based on previous experiments for Aβ induction in APP_swe_/PS1_dE9_ mice after inoculation of human brain extracts (estimated with significance level of 5%, a power of 80%, and a two-sided test) [12] and increased to take into account uncertainties for new markers (memory and synaptic changes). No animals were excluded from the study. MC was aware of initial group allocation, but further analyses (memory evaluations and post-mortem studies) were performed blindly.

### Stereotaxic surgery

Five-hundred micrograms/ml (~ 150 nM) of Aβ_wt_, Aβ_osa_ solution were rapidly thawed out before stereotaxic injection. Two month-old APP_swe_/PS1_dE9_ and wild-type littermates were anesthetized by an intraperitoneal injection of ketamine (1 mg/10 g; Imalgène 1000, Merial) and xylazine (0.1 mg/10 g; 2% Rompun, Bayer Healthcare). Local anesthesia was also performed by a subcutaneous injection of lidocaine at the incision site (1 µL/g; 0.5% Xylovet, Ceva). Mice were placed in the stereotaxic frame (Phymep) and bilateral injections of brain samples were performed in the dentate gyrus (AP -2 mm, DV 1.8 mm, L ± 2 mm from bregma). Two µl/site of sample were administered using 34-gauge needles and Hamilton syringes, at a rate of 0.2 µl/min. After the injection, needles were kept in place for 5 more minutes before removal and the incision was sutured. The surgical area was cleaned before and after the procedure using povidone iodine (Vétédine, Vétoquinol). Respiration rate was monitored and body temperature was maintained at 37 ± 0.5 °C with a heating pad during the surgery. Anesthesia was reversed with a subcutaneous injection of atipamezole (0.25 mg/kg; Antisedan, Vetoquinol). Mice were placed in a ventilated heating box (25 °C) and monitored until full recovery from anesthesia. Postoperative anticipatory pain management consisted of paracetamol administration in drinking water (1.45 mL/20 mL of water; Doliprane, Sanofi) during 48 h.

### Behavioral evaluations

A novel object recognition task in a V-maze was used to investigate cognition at 4 mpi on Aβ_wt-_, Aβ_osa_-, and PBS-inoculated APP_swe_/PS1_dE9_ mice. Wild-type littermates injected with PBS were used as controls for the tests. Mice were handled for 2 min per day, for 5 days prior to any test to prevent stress effects during tasks. Prior to each test, mice were habituated to the experimental room for 30 min. The experimenter was blind to mouse groups. Performances were recorded using a tracking software (EthoVision XT14, Noldus).

The V-maze arena consisted of two 6 cm-wide, 33.5 cm-long and 15 cm-high black arms forming a V shape and exposed to 50 lx-lighting. The test was divided into three phases, each one separated by 24 h. At the beginning of each session, mice were placed at the center of the arena, *i.e.* at the intersection of the arms. During the habituation phase (day 1), mice were free to explore the empty arena for 9 min. The distance traveled was automatically recorded as an indicator of their exploratory activity. For the training phase (day 2), two identical objects (bicolor plastic balls) were placed at the end of each arm. Exploratory activity was evaluated as the time spent exploring the objects (manually recorded) and the distance traveled during the 9-min trial. On the test day (day 3), one familiar object (a bicolor plastic ball) was replaced by a novel one of a different shape and material (a transparent glass flask). Recognition was assessed using a discrimination index, calculated as follows:

$${\text{Discrimination}}\;{\text{index}} = \frac{{{\text{Time}}\;{\text{exploring}}\;{\text{the}}\;{\text{novel}}\;{\text{object}} - {\text{Time}}\;{\text{exploring}}\;{\text{the}}\;{\text{familiar}}\;{\text{object}}}}{{{\text{Total}}\;{\text{exploration}}\;{\text{time}}}}$$

It reflects the time spent exploring each object, and therefore, the ability to discriminate a novel object from a familiar, previously explored one. A low discrimination index score reveals that mice spent less time exploring the new object, *i.e.* still had marked interest in the familiar object, and suggests that memory was impaired. Between each run, the V-maze was cleaned with 10% ethanol, effectively eliminating any scents from previous visits.

### Animal preparation and MRI acquisition

To further evaluate brain function, we performed resting-state functional magnetic resonance imaging studies (fMRI) at 4 mpi. Our objective was i. to analyze the strength of the hippocampus functional connectivity (FC) to the whole-brain, ii. to analyze the FC of specific regions of the hippocampus and of the entorhinal cortex to the whole-brain. Animals were scanned four months post-inoculations. Acquisitions were performed on anesthetized mice with a combination of isoflurane 0.5% in a mix 0.5:0.5 of air:O_2_ and medetomidine 0.3 mg/kg bolus and 0.1 mg/kg/h infusion. Animals were freely-breathing and respiratory rate was monitored to confirm animal stability until the end of the experiment. Body temperature was maintained by a water heating system at 37 °C. The MRI system was an 11.7 Tesla Bruker BioSpec (Bruker, Ettlingen, Germany) using a Cryoprobe surface and running ParaVision 6.0.1. First, anatomical images were acquired using a T2-weighted multi-slice multi-echo (MSME) sequence: TR = 1000 ms, TE = 5 ms, 6 echoes, inter-echo time = 5 ms, FOV = 16 × 16 mm, 100 slices of 0.2 mm thickness, resolution = 200 µm isotropic, acquisition duration 10 min. Then, resting-state functional MRI (rs-fMRI) was acquired using a gradient-echo echo planar imaging (EPI) sequences with repetition time (TR) = 1000 ms, echo time (TE) = 10 ms, flip angle = 90°, volumes = 500, Field of view (FOV) = 22.5 × 15.4 mm, 12 slices of 0.5 mm thickness, acquisition duration 7 min 30 s. Animals were scanned twice with this sequence. For analysis, the two scans were merged.

### MRI pre-processing and analysis

Scanner data were exported as DICOM files then converted into NIfTI-1 format. Then spatial pre-processing was performed using the python module sammba-mri (SmAll MaMmals BrAin MRI; http://sammba-mri.github.io, [3]). First, spatial normalization of the anatomical images was performed to generate a high-resolution template. Rs-fMRI images were corrected for slice timing (interleaved), motion, and B0 distortion (per-slice registration to respective anatomicals). Then, rs-fMRI images were co-registered to the high-resolution template of the DSURQE anatomical atlas (Dorr-Steadman-Ulman-Richards-Qiu-Egan (DSURQE) atlas, 182 structures, freely available at: https://wiki.mouseimaging.ca/display/MICePub/Mouse+Brain+Atlases) [4, 34, 42, 48]. Functional images were further pretreated using Nilearn [1]. Nuisance signal regression was applied including a linear trend as well as 24-motion confounds (6 motion parameters, those of the preceding volume, plus each of their squares [10]). Images were then spatially smoothed with a 0.4 mm full-width at half-maximum Gaussian filter. The first 10 volumes were excluded from analysis after the preprocessing to ensure steady-state magnetization.

### MRI analysis and statistics

We analyzed the strength of the hippocampus functional connectivity (FC) to the whole-brain. First, we quantified the whole-brain pattern of functional connectivity for each subject by computing Pearson’s correlation coefficients between mean time series of 70 bilateral regions defined from a brain atlas. The average correlation coefficient between the hippocampus and all other brain areas was then evaluated. Second, we evaluated FC of three selected brain regions (right hemisphere) to the whole-brain: the MoDG (inoculation site), the CA1 area (which contains the pyramidal cell layer (CAPy)) and the entorhinal cortex. We used a “seed-based analysis” (SBA) to identify regions functionally associated with each selected brain region. This method assesses the connection between cluster of voxels (called seeds) positioned in the brain. Here the seeds (in MoDG, CAPy or entorhinal cortex) were defined based on the DSURQE Dorr atlas using 0.3 mm^3^ spheres, corresponding to 25 voxels. The mean BOLD signal time-series within a seed were extracted and regressed into individual scans to obtain correlation z-statistic maps using Nilearn. Voxelwise statistics were carried out in FSL using nonparametric permutation tests (randomize) for comparison between PBS- inoculated APP_swe_/PS1_dE9_ and either Aβ_wt_- or Aβ_osa_-inoculated APP_swe_/PS1_dE9_ (5000 permutations and voxelwise correction). 3D representation of voxelwise statistical maps are shown as color-coded t-statistics overlays on the DSURQE template at 0.04 × 0.04 × 0.04 mm^3^ using MRIcroGL (https://www.nitrc.org/projects/mricrogl/).

### Animal euthanasia and brain preparation

Mice were sacrificed at 4 mpi, after MRI acquisition, with an intraperitoneal injection of a lethal dose of pentobarbital (100 mg/kg; Exagon, Axience). They were perfused intracardially with cold sterile 0.1 M PBS for 4 min, at a rate of 8 ml/min. The brain was extracted and separated in two hemispheres. The left hemisphere was dissected in order to take out the hippocampus and the cortex. Samples were directly deep-frozen into liquid nitrogen and stored at − 80 °C for biochemical analysis. For histology, the right hemisphere was post-fixed in 4% paraformaldehyde for 48 h at + 4 °C, transferred into a 15% sucrose solution for 24 h and in a 30% sucrose solution for 48 h at + 4 °C for cryoprotection. Serial coronal sections of 40 µm were performed with a microtome (SM2400, Leica Microsystem) and stored at −2 °C in a storing solution (glycerol 30%, ethylene glycol 30%, distilled water 30%, phosphate buffer 10%).

### Mouse brain sample preparation for biochemical analyses

For amyloid protein extraction, deep-frozen brain tissue was dissociated with Collagenase D (2 mg/mL) in Tris-buffer saline (20 mM Tris–HCl, 150 mM NaCl, pH 7.4) at 1:10 TBS volume:brain wet weight and incubated at 37 °C. Brains were further homogenized using a Dounce homogenizer with 20 strokes in ice-cold quench buffer containing protease inhibitors (Complete, PMSF 1 mM) and phosphatase inhibitors (Na_3_VO_4_ 1 mM, NaF 10 mM). Sarkosyl (2%) were added to homogenates. Samples were centrifuged for 30 min at 10,000 × g at 4 °C. The resulting supernatant was further centrifuged for 1 h at 100,000 g in a 4 °C TLA100.2 rotor on Beckman TL 100. The resulting supernatant called S100K contained the sarkosyl-soluble fraction. Pellet (P100K) was washed twice in TBS and finally resuspended in TBS at 1:10 TBS volume:brain wet weight. All samples were stored at −80 °C until analysis.

### Western blotting and dot blotting

For western blot, samples were diluted in LDS (Lithium Dodecyl Sulfate) sample buffer (NuPage) and sampling reducing agent in order to load 20 µg of proteins. After heating, samples were loaded on a 4–12% Criterion™ XT Bis–Tris gel (Bio-Rad), migrated in XT MES Running Buffer (Bio-Rad) for 1 h at 110 V and transferred onto 0.2 µm nitrocellulose. For dot blot, 2µL of samples were directly loaded onto 0.2 µm nitrocellulose. After 1 h of blocking at room temperature, membranes were blotted overnight at 4 °C with primary antibodies 6E10 (Against human Aβ_1-16_; Biolegend), APP-Cter-17 (against the last 17 amino acids of the human APP sequence [39, 51], A11 (against oligomeric species, ThermoFisher) and OC (anti-amyloid fibrils, Rockland). After rinse in TBS-T, membranes were incubated with secondary antibodies for 1 h at room temperature. Proteins were revealed using horseradish peroxidase (HRP) and ECL™ Western Blotting Detection Reagent (G&E Healthcare). Quantifications of protein expression levels were performed on ImageJ Software.

### Immunohistochemistry

Aβ deposits were evaluated using a 4G8 labeling. Free-floating brain sections were rinsed in a 0.1 M PBS solution (10% Sigma-Aldrich® phosphate buffer, 0.9% Sigma-Aldrich® NaCl, distilled water) before use. Washing and incubation steps were performed on a shaker at room temperature unless indicated otherwise.

4G8 labeling was performed after pretreating brain sections with 70% formic acid (VWR®) for 20 min at room temperature. All tissues were then incubated in 30% hydrogen peroxide (Sigma-Aldrich®) diluted 1/100 for 20 min to inhibit endogenous peroxidases. Blocking of non-specific antigenic sites was achieved over 1 h using a 0.2% Triton X-100/0.1 M PBS (Sigma-Aldrich®) (PBST) solution containing 4.5% normal goat serum or 5% bovine serum albumin. Sections were then incubated at + 4 °C with the 4G8 (Biolegend 800,706, 1/500) antibody diluted in a 3%NGS/PBST solution for 48 h. After rinsing, an incubation with the appropriate biotinylated secondary antibody diluted to 1/1000 in PBST was performed for 1 h at room temperature, followed by a 1 h incubation at room temperature with a 1:250 dilution of an avidin–biotin complex solution (ABC Vectastain kit, Vector Laboratories®). Revelation was performed using the DAB Peroxidase Substrate Kit (DAB SK4100 kit, Vector Laboratories®). Sections were mounted on Superfrost Plus slides (Thermo-Scientific®). All sections were then dehydrated in successive baths of ethanol at 50°, 70°, 96° and 100° and in xylene. Slides were mounted with the Eukitt® mounting medium (Chem-Lab®).

Stained sections were scanned using an Axio Scan.Z1 (Zeiss®—Z-stack images acquired at 20 × (z-stacks with 16 planes, 1 µm steps with extended depth of focus)). Each slice was extracted individually in the "czi" format using the Zen 2.0 (Zeiss®) software. Image processing and analysis were performed with the ImageJ 1.53i software. Macros were developed for each staining in order to attain a reproducible semi-automated quantification. Images were imported with a 50% reduction in resolution (0.44 µm/pixel), converted to the RGB format and saved as "tif". The 4G8 immunostaining was highlighted by color deconvolution with the H DAB vectors and by selecting the resulting DAB image. Then, segmentation was performed through an automatic local thresholding using the Phansalkar method (radius = 50). Aβ load was evaluated after quantification of the 4G8-labeled particles between 7 and 5,000 µm^2^, and normalization to the surface area of each region of interest (ROI).

All quantifications were performed on adjacent slices between 1.98 and −4.36 mm from bregma. Eighteen adjacent slices were analyzed for the 4G8. All ROIs were manually segmented using ImageJ/FIJI, according to the Paxinos and Franklin neuro-anatomical atlas of mouse brain [33].

### Evaluation of synaptic density

Synaptic density was evaluated in the hippocampus (CA1) and the perirhinal/entorhinal cortex of all inoculated mice using a double immunolabeling of presynaptic (Bassoon) and postsynaptic (Homer1) markers. Free-floating sections were permeabilized in a 0.5% Triton X-100/0.1 M PBS (Sigma-Aldrich®) solution for 15 min. Slices were incubated with Bassoon (Abcam Ab82958, 1/200) and Homer1 (Synaptic systems 160,003, 1/400) antibodies diluted in 3% BSA/PBST solution for 24 h at RT. Incubation with secondary antibodies coupled to a fluorochrome (Alexa Fluor) diluted in a 3% BSA/0.1 M PBS solution was then performed for 1 h at room temperature. Sections were rinsed and mounted on Superfrost Plus (Thermo-Scientific®) slides with the Vectashield® mounting medium with a refractive index of 1.45. Images of stained sections were acquired using a Leica DMI6000 confocal optical microscope (TCS SPE) with a 40 × oil-immersion objective (refractive index 1.518) and the Leica Las X software. A confocal zoom of 3 and a pinhole aperture fixed at 1 Airy were applied. Acquisition was performed in sequential mode with a sampling rate of 1024 × 1024 and a scanning speed of 700 Hz. Image resolution was 80 nm/pixel and the optical section was 0.896 µm. 26 separate planes with a 0.2 µm step were acquired. The excitation wavelengths were 594 nm or 633 nm. Image acquisition in the dentate gyrus, CA1 and CA2/3 region was performed on 5 adjacent slices located between −1.58  and −3.40 mm from the bregma, with 3 images per slice. For the entorhinal cortex, 3 adjacent slices located between −2.98 and −3.40 mm from the bregma were analyzed, with 2 images acquired per slice. 3D deconvolution of the images was performed using the AutoQuant X3 software. The deconvoluted 8-bit images were analyzed using the ImageJ software, as described in Gilles et al. [13]. Briefly, automated 3D segmentation of the presynaptic (Bassoon) and postsynaptic (Homer1) stained deconvoluted images was performed using "3D spots segmentation" from ImageJ (with "gaussian fit", "block" and "no watershed" options; https://imagej.net/plugins/3d-segmentation). Co-localization of overlapping objects was evaluated using "DiAna" from imageJ (https://imagej.net/plugins/distance-analysis). The percentage of colocalized objects was quantified as an index of synaptic density.

### Statistical analysis

Statistical analysis was performed using the GraphPad Prism software 9. For the behavioral tasks analysis, Kruskal–Wallis tests with Dunn’s multiple comparisons were performed except when repeated measures were acquired, in which case, a two-way repeated measures ANOVA with the Geisser-Greenhouse correction and Dunnett’s multiple comparisons was carried out. For the post-mortem analysis, Kruskal–Wallis tests with Dunn’s multiple comparisons tests were performed in order to compare differences between inoculated mice. The significance level was set at *p* < 0.05. Data are shown on scattered dot plots with mean ± standard error of the mean (SEM).

### Data availability

The data that support the findings of this study are available from the corresponding author, upon request.

## Result

### Characterization of inoculated Aβ peptides: from structures to synapse-modifying properties

**Fig. 1:**
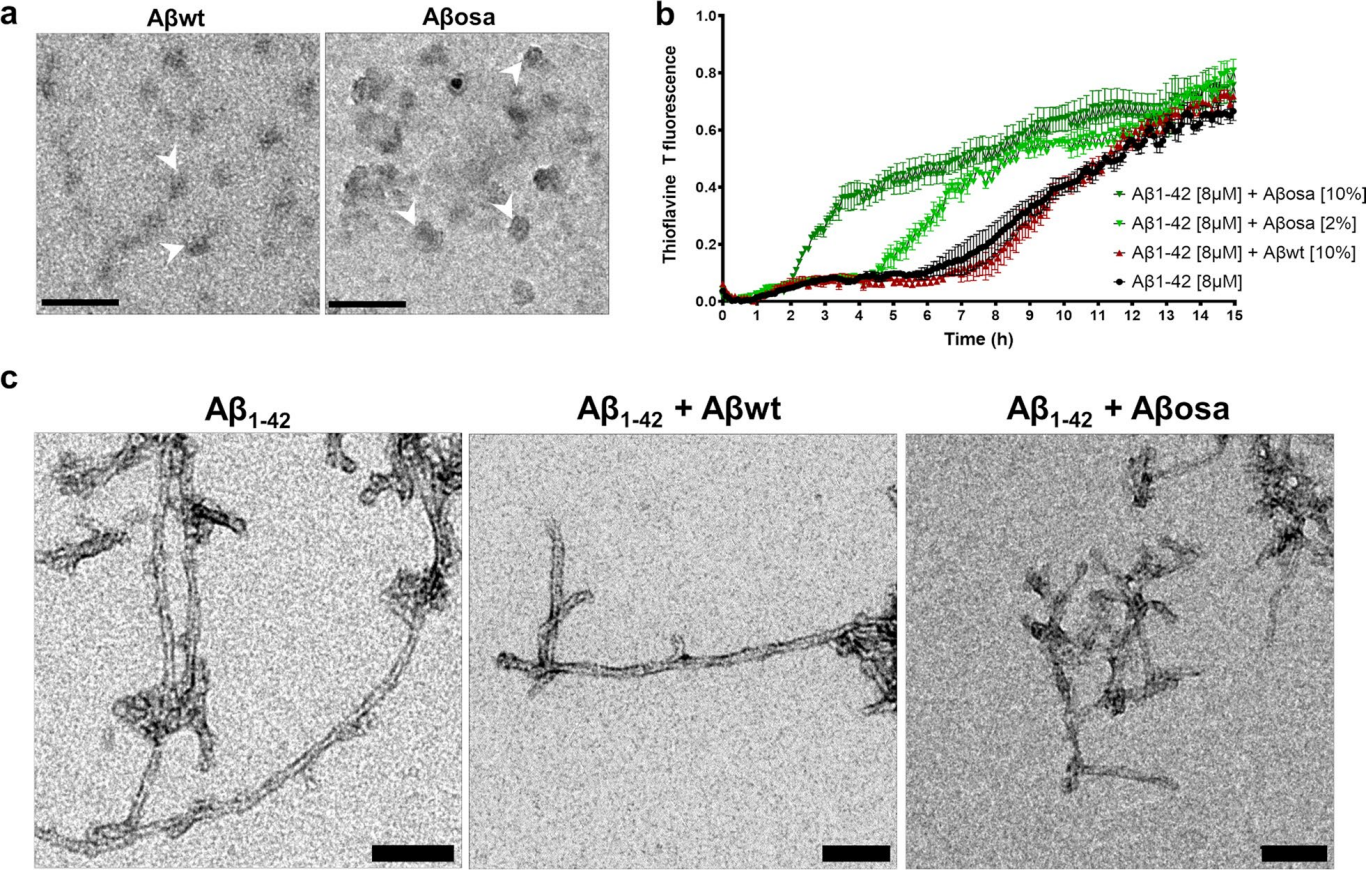
Properties of wt and mutated Aβ assemblies. **a.** Representative electron microscopy images of small and large particles in Aβ_wt_ and Aβ_osa_ solution. Scale bars: 100 nm. **b.** Kinetics of synthetic Aβ_1-42_ aggregation monitored by thioflavin T fluorescence in the absence and presence of Aβ_wt_ and Aβ_osa_ seeds. Aggregation experiments were performed in triplicates. Aβ_1-42_ monomer concentration is 8 μM, in a PBS buffer at 37 °C with continuous agitation. The aggregation curves were normalized to maximal values of ThT fluorescence at plateau. Symbols and error bars are the average and standard deviation, respectively, of three independent kinetics experiments. **c.** Representative electron microscopy images Aβ fibrils following the aggregation experiments. Scale bars: 100 nm

We used a recombinant approach to produce non-mutated Aβ_osa_ (E693Δ also called E22Δ) or Aβ_1-42_ (Aβ_wt_). Electron micrographs showed that both Aβ peptides were able to self-aggregate and formed Aβ assemblies in solutions (Fig. 1a). Aβ aggregation is a nucleation-dependent polymerization process, with a slow initial nucleation phase, called lag-phase, followed by a rapid growth phase [18]. We investigated the seeding properties of different forms of Aβ in vitro using thioflavin fluorescence assay [27, 32]. First, synthetic monomeric Aβ_1-42_ was incubated at 37 °C, the ThT fluorescence signal displayed a sigmoidal shape characterized by a 6 h lag time followed by an 8 h elongation step (Fig. 1b). When synthetic monomeric Aβ_1-42_ was seeded with recombinant Aβ_wt_ [10%, v/v] at 37 °C, assembly kinetic was not affected while Aβ fibrils were formed (Fig. 1b). On the contrary, addition of Aβ_osa_ seeds [2 and 10%, v/v] shortened the lag time to 2 and 4 h respectively (Fig. 1b). Thus, Aβ_osa_ displayed increased seeding effects. At steady state, the Aβ fibrils assembled from synthetic Aβ_1-42_ alone or seeded with Aβ_wt_ or Aβ_osa_ displayed undistinguishable shape when observed by electron microscopy (Fig. 1c).

### Exogenous application of Aβ_osa_ and Aβ_wt_ impairs synaptic plasticity and induces spine losses

**Fig. 2:**
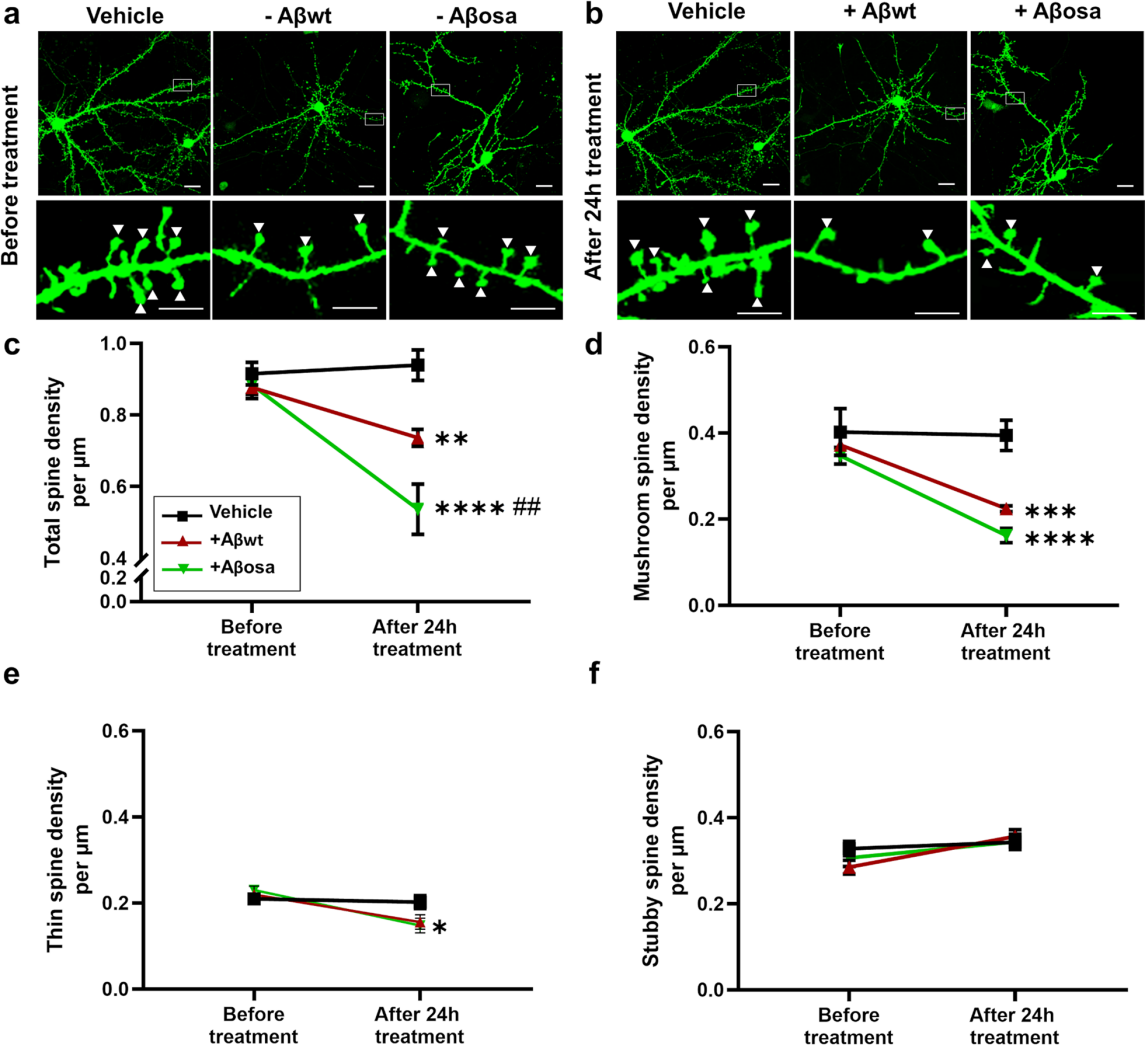
Exposure to exogenous Aβ_osa_ and Aβ_wt_ differentially impaired synapses. **a-b.** Representative images of primary cultures of cortical neurons expressing LA-GFP before (a), and after treatment for 24 h with 100 nM of Aβ_osa_ or Aβ_wt_ (b). Top row wide field view, scale bar = 10 µm; bottom row: dendrite portions with mushroom spines (white arrows, scale bar = 5 µm). **c.** Quantification of total spine density showed a reduction of total number of spines after treatment with Aβ_osa_ and Aβ_wt_, compared to PBS (34.7 ± 3.1% for Aβ_osa_, p < 0.0001; 14.1 ± 3.4% for Aβ_wt_, p = 0.0049). The spine loss was significantly more severe in Aβ_osa_ compared to Aβ_wt_ (*p* = 0.0048). **d.** Quantification of mushroom spine density showed a reduction of the number of mushroom spines after treatment with Aβ_wt_ and Aβ_osa_ (respectively *p* = 0.0005 and *p* < 0.0001). **e.** Quantification of thin spine density showed a reduction of the number of mushroom spines after treatment with Aβ_osa_ (*p* < 0.1). **f.** Stubby spine density was not modified after treatment with the different Aβ seeds. *n* = 6 neurons from at least 3 different cultures. Data are shown as mean ± s.e.m. Kruskal–Wallis with Dunn’s multiple comparisons. *p < 0.5, ** or ## *p* < 0.05, ***p < 0.005, ****p < 0.0005

As Aβ has been shown to induce synaptic dysfunction [22], we compared the direct impact of Aβ_osa_ and Aβ_wt_ seeds on synaptic health by assessing spine morphology of primary cortical neuron cultures. Cortical neurons were co-transfected with LifeActin-GFP (LA-GFP), a small peptide that specifically binds to F-actin to visualize the dendritic arbor and spines. LA-GFP expressing cortical neurons display characteristic dendrites with a high density of spines (Fig. 2a). Then, we incubated the neurons with 100 nM of either Aβ_osa_ or Aβ_wt_ for 24 h, and analyzed thin, stubby, mushroom spine density (Fig. 2b-f). Compared to PBS, the total spine density of neurons treated with Aβ_osa_ or Aβ_wt_ was reduced at 24 h (*p* < 0.0001 for Aβ_osa_; p < 0.05 for Aβ_wt_, Fig. 2c). The spine loss was significantly more severe in Aβ_osa_ compared to Aβ_wt_ (*p* < 0.05, Fig. 2c). The spine loss was mainly due to a strong reduction in mushroom (*p* < 0.0001 for Aβ_osa_; *p* < 0.005 for Aβ_wt,_ Fig. 2d) and thin spine densities (p < 0.5 for Aβ_osa,_ Fig. 2e) while stubby spine density was not significantly affected by Aβ compared to PBS (Fig. 2f).

### Aβ_osa_ inoculation to rodents impairs long-term memory

**Fig. 3:**
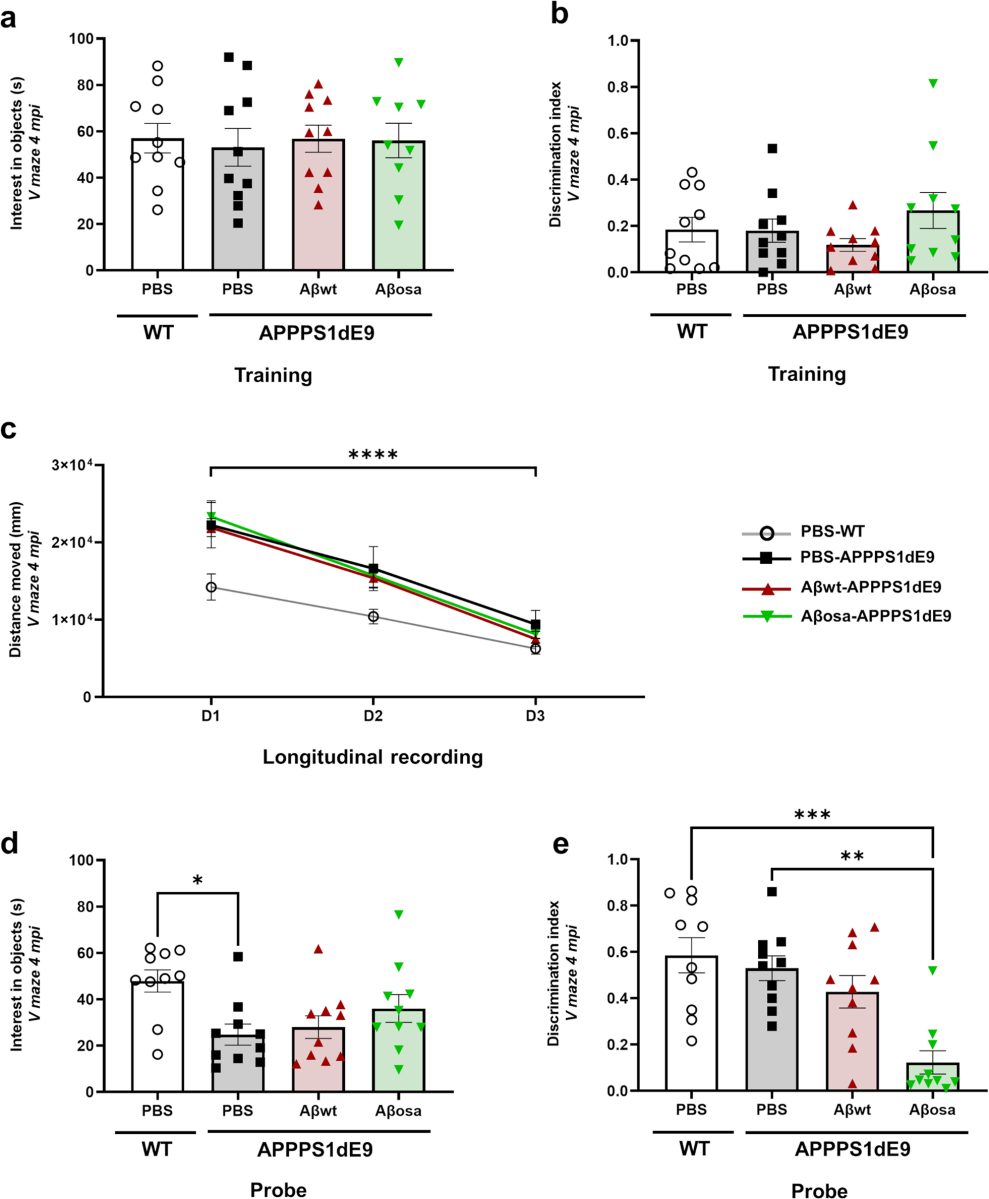
Memory impairment of APP_swe_/PS1_de9_ mice following Aβ inoculation. Novel object recognition was evaluated in a V-maze at 4 months post-inoculation. **a.** The time spent on exploring the two identical objects (in seconds) during the training phase is similar between group (p = 0.94). **b.** Mice performance during the training phase. Similar discrimination indexes were found for all groups when mice had to discriminate two identical objects (p > 0.05, Kruskal–Wallis with Dunn’s multiple comparisons). **c.** Distance moved throughout 3 days of tests (exploration, training and probe-test days). Measurements revealed a time-effect from day 1 to day 3 (F_(1.49, 53.95)_ = 123.8, *p* < 0.0005), but no differences between experimental groups (*p* > 0.05) (two-way repeated measures ANOVA with the Geisser-Greenhouse correction and Dunnett’s multiple comparisons). **d.** Novel object recognition evaluated by the time spent on exploring the objects (in seconds) highlighted group effects (*p* = 0.02). Post-hoc analysis showed that the PBS-inoculated wild-type mice group had a higher exploratory activity than PBS-inoculated APP_swe_/PS1_dE9_ (*p* = 0.02) while all groups of APP_swe_/PS1_de9_ mice had comparable exploratory activity (Kruskal–Wallis with Dunn’s multiple comparisons).** e.** Object discrimination index. APP_swe_/PS1_de9_ mice inoculated with Aβ_osa_ spent less time exploring the novel object compared to PBS-inoculated WT mice or APP_swe_/PS1_dE9_ mice (group analysis using Kruskal–Wallis (*p* = 0.0006) with post-hoc analysis using Dunn’s multiple comparisons *p* = 0.0008 and *p* = 0.0046, for Aβ_osa_ versus PBS-inoculated WT and APP_swe_/PS1_dE9_ mice, respectively). n_WT-PBS_ = 10, n_APP/PS1-PBS_ = 10, n_Aβwt_ = 10, n_Aβosa_ = 10 mice. Data are shown as mean ± s.e.m

We then evaluated the long-term impact of Aβ in vivo. Two-month-old APP_swe_/PS1_de9_ mice were inoculated bilaterally with 0.5 µg of Aβ_osa_ or Aβ_wt_ or PBS in the molecular layer of the dentate gyrus (MoDG) of the hippocampus. PBS was also inoculated in wild-type (WT) littermates. Behavioral assessment was performed at 4 months post-inoculation (mpi) using a novel object recognition task in a V-maze test (Fig. 3). First, the mice underwent a habituation to the test in an empty arena (day 1) followed by a training phase where two identical objects were added to the V-maze platform (day 2). Comparable interest in objects (Fig. 3a) and discrimination index (Fig. 3b) were observed during the training phase indicating similar exploratory activity between groups. The probe test (day 3) was performed the day following the training phase. During this task, a novel object replaced one of the objects. The traveled distance decreased throughout the three days of the test, but was comparable between groups (Fig. 3c). During the probe test, all groups of APP_swe_/PS1_de9_ mice had comparable interest in objects (Fig. 3d). During the discrimination task, mice inoculated with Aβ_osa_ spent less time exploring the novel object compared to control mice (PBS-inoculated WT or APP_swe_/PS1_dE9_ mice), suggesting memory impairments (Fig. 3e).

### Aβ_osa_ inoculation leads to long-term functional impairments

**Fig. 4:**
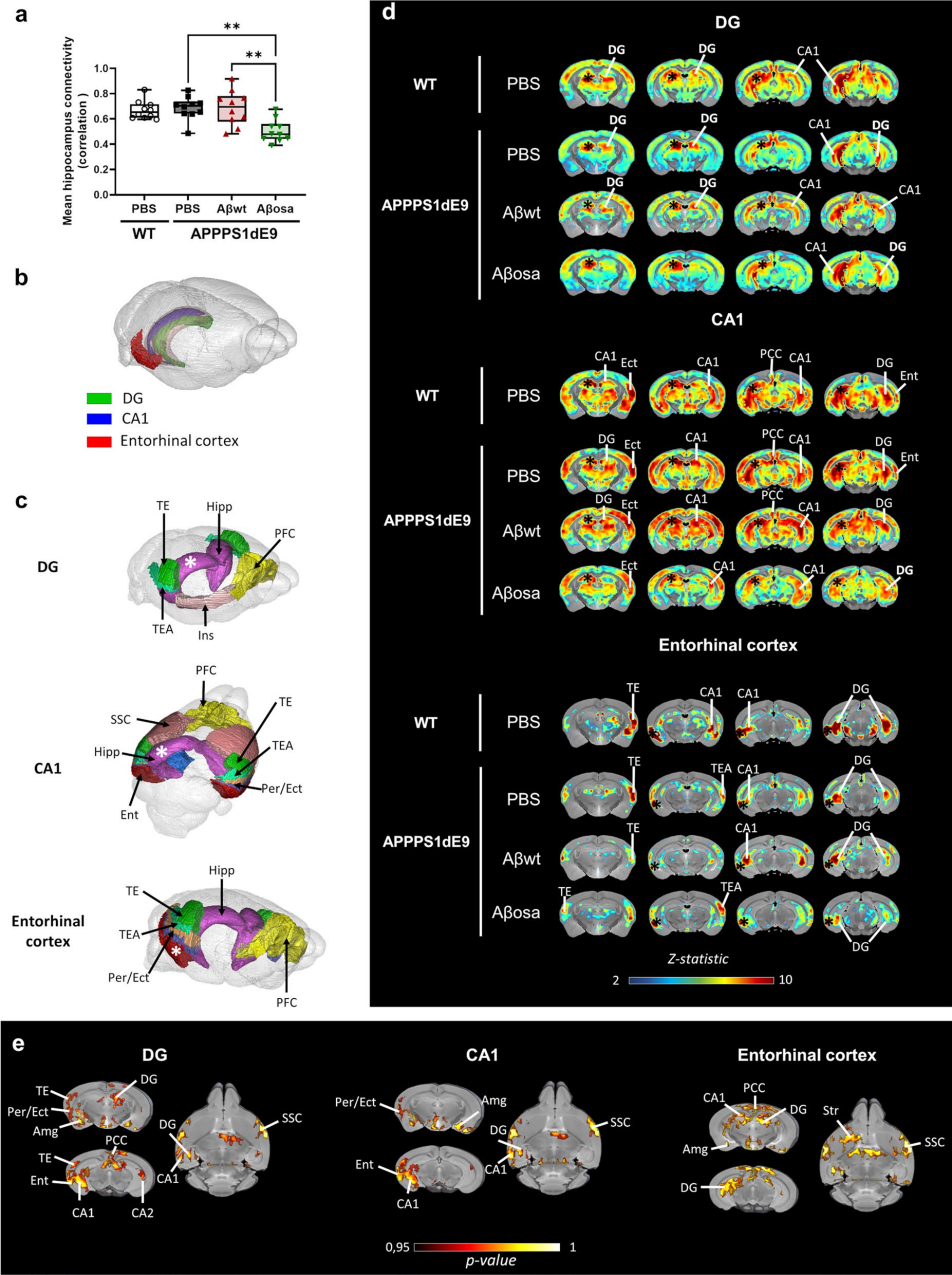
Aβ_osa_ inoculated animals displayed abnormal brain connectivity within hippocampal-memory circuits. **a.** Mean hippocampus connectivity changes measured by resting-state functional MRI. Aβ_osa_-inoculated group displayed a decreased hippocampus connectivity compared to PBS and Aβ_wt_-inoculated mice at 4mpi (Kruskal–Wallis with Dunn’s multiple comparisons. group effect *p* = 0.002; *p* = 0.006 and *p* = 0.01 for Aβ_osa_-inoculated group versus PBS or Aβ_wt_-inoculated mice). **b.** 3D representation of the three brain regions -DG, CA1 and the entorhinal cortex- used for the seed-based analyses (SBA). **c.** SBA-derived resting state networks found in PBS-inoculated APP_swe_/PS1_dE9_ are shown for each seed (the white asterisk represents the location of the seeds where the signal was extracted). **d.** Correlation maps of each seed. The color scale bar represents the strength of the functional correlation normalized with a fisher z-transformation. Black asterisks represent the location of the seeds. Differences are found between groups in inter-hemispheric homotopic FC. **e.** Voxelwise nonparametric permutation tests of FC correlation maps. Aβ_osa_-inoculated mice have a lower FC in the hippocampus compared to PBS-inoculated mice (*p* < 0.001). The color scale bar represents the statistical significant p-value. DG = dentate gyrus, Hipp = hippocampus, TE = temporal area, TEA = temporal associative area, Per/Ect = perirhinal + ectorhinal cortex, Ins = Insular, Amg = amygdalar area, Ent = entorhinal cortex, PCC = posterior cingulate cortex, SSC = primary somatosensory area, Str = striatum. n_WT-PBS_ = 10, n_APP/PS1-PBS_ = 10, n_Aβwt_ = 10, n_Aβosa_ = 10 mice

To further evaluate brain function, we performed resting-state functional magnetic resonance imaging studies (fMRI) at 4 mpi. First, we analyzed the strength of the hippocampus functional connectivity (FC) to the whole-brain. The correlation coefficient between the hippocampus and other brain areas was lower in Aβ_osa_-inoculated animals compared to PBS and Aβ_wt_-inoculated mice (Fig. 4a). This indicates a decreased hippocampus connectivity in Aβ_osa_-inoculated mice. To further identify specific networks impaired by Aβ_osa_ inoculation, we evaluated FC of three selected brain regions (right hemisphere) to the whole-brain: the MoDG (inoculation site), the CA1 area (which contains the pyramidal cell layer (CAPy)) and the entorhinal cortex (Fig. 4b). First we used a “seed-based analysis” (SBA), to identify regions functionally associated with each selected brain region in the PBS-inoculated APP_swe_/PS1_dE9_ mice (Fig. 4c). The individual correlation maps were averaged for each group to highlight connected areas in each group of animals (Fig. 4d). Homotopic functional connectivity of MoDG was abolished in the contralateral dorsal dentate gyrus of Aβ_osa_-inoculated mice while unaffected in Aβ_wt_-inoculated animals (Fig. 4d). The MoDG and the CA1 of Aβ_osa_-inoculated mice had also lower FC with the peri-hippocampal area, amygdalar area, temporal area and ventral CA1. The entorhinal cortex of Aβ_osa_-inoculated mice had lower FC with the posterior cingulate, amygdala areas and the hippocampus area compared to PBS-inoculated mice (Fig. 4d). The alterations reported in Aβ_osa_ animals concerned both left-to-right (displayed figures) and right to left connectivities (not shown).

The individual correlation maps between groups were compared by performing voxel-wise statistical analysis. They confirmed differences between Aβ_osa_- and PBS-inoculated APP_swe_/PS1_dE9_ mice (Fig. 4e) and did not show differences between Aβ_wt_- and PBS-inoculated mice **(not shown)**. The FC alterations in Aβ_osa_-inoculated animals that involve memory circuits are consistent with cognitive impairments reported in transgenic animals expressing Aβ_osa_ variant.

### Aβ_osa_ inoculation reduces synaptic density

**Fig. 5:**
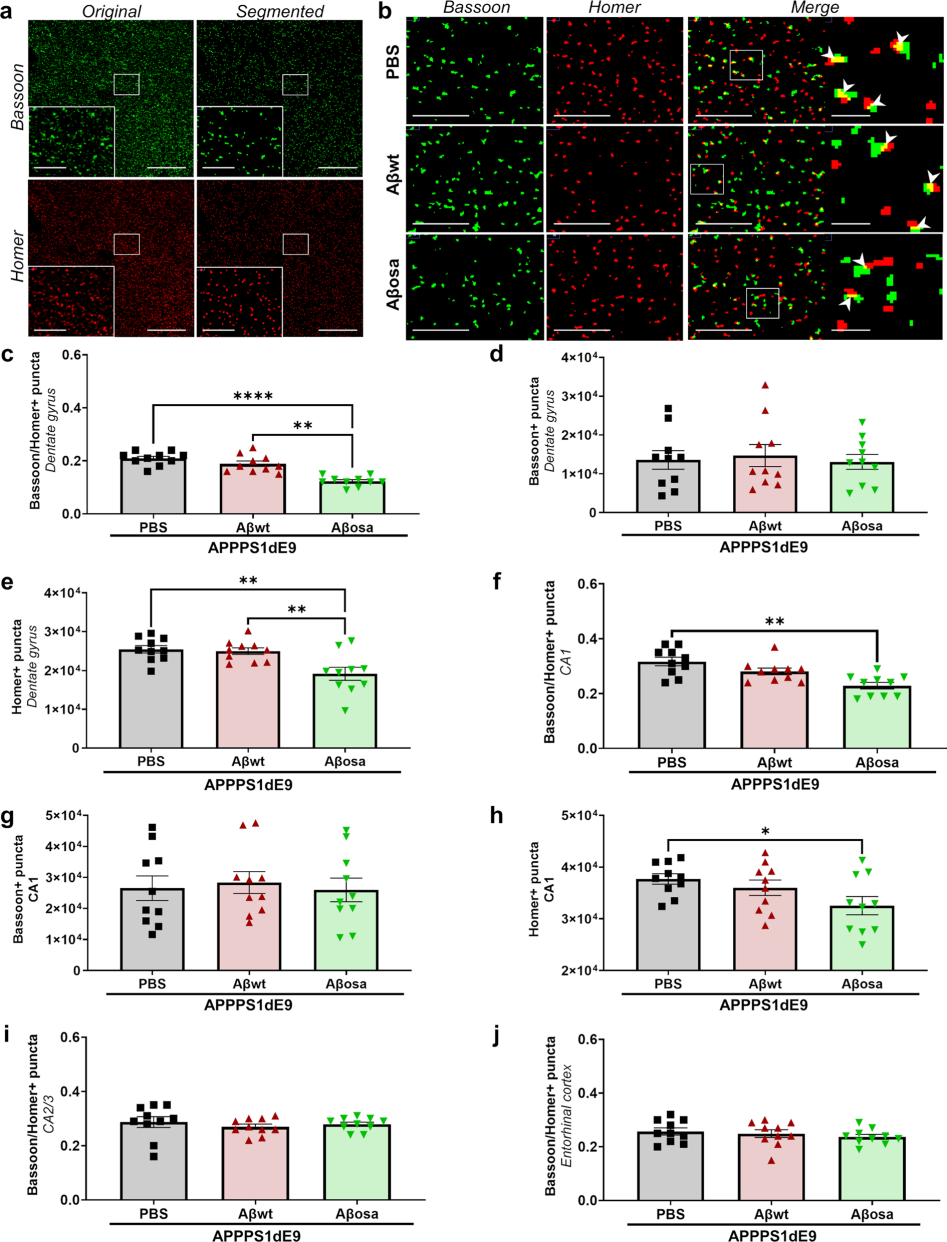
Aβ_osa_ exacerbates long-term synaptotoxicity in vivo.** a.** Representative views of original Bassoon/Homer images and segmented puncta in APP_swe_/PS1_dE9_ mice. Scale bars main images: 20 µm; Insets: 5 µm. **b.** Co-localisation puncta of Bassoon/Homer labels (white arrow). Scale bars main images: 5 µm; Insets: 1 µm. **c-h.** Quantification of synaptic density from Bassoon/Homer colocalization (**c, f**), Bassoon (**d, g**) and Homer (**e, h**) in the dentate gyrus and and CA1 showed decrease of synaptic density and post-synaptic density (Homer) in the dentate gyrus and CA1 of Aβ_osa_-inoculated APP_swe_/PS1_dE9_ mice (**c**: Bassoon/Homer in dentate gyrus—overall effect: p < 0.0001 (Kruskal–Wallis). Post-hoc evaluation with Dunn’s multiple comparisons: Aβ_osa_- versus PBS-inoculated APP_swe_/PS1_dE9_: *p* < 0.0001; Aβ_wt_- versus Aβ_osa_-inoculated APP_swe_/PS1_dE9_: p = 0.003; **e**: Homer in dentate gyrus—overall effect: *p* = 0.0015 (Kruskal–Wallis). Post-hoc evaluation with Dunn’s multiple comparisons: Aβ_osa_- versus PBS-inoculated APP_swe_/PS1_dE9_: p = 0.0029; Aβ_wt_- versus Aβ_osa_-inoculated APP_swe_/PS1_dE9_: *p* = 0.0058; **f**: Bassoon/Homer in CA1—overall effect: *p* = 0.002 (Kruskal–Wallis). Post-hoc evaluation with Dunn’s multiple comparisons: Aβ_osa_- versus PBS-inoculated animals: *p* = 0.002; **h**: Homer in CA1—overall effect: *p* = 0.0535 (Kruskal–Wallis). Post-hoc evaluation with Dunn’s multiple comparisons: Aβ_osa_- versus PBS-inoculated APP_swe_/PS1_dE9_: *p* = 0.0469. There were no changes in the different groups in the CA2/3 (**i**) and in the entorhinal cortex (**j**). n_APP/PS1-PBS_ = 10, n_Aβwt_ = 10, n_Aβosa_ = 10 mice. For each image, quantification was made on 26 sections separated by 0.2 µm step. A surface of 81.92*81.92µm^2^ was measured for each section. Data are shown as mean ± s.e.m

Mice were sacrificed 4 mpi to evaluate cerebral pathology. We measured synaptic density at the inoculation site (dentate gyrus of the hippocampus), in other parts of the hippocampus and at a distant place (entorhinal cortex). Double immunolabeling of presynaptic (Bassoon) and postsynaptic (Homer) markers was performed (Fig. 5a–b) and the amount of synapses was quantified from colocalized puncta. Inoculation of Aβ_osa_ led to decreased synaptic density in the dentate gyrus (Fig. 5c–e) and CA1 region (Fig. 5f–h) compared to APP_swe_/PS1_dE9_ mice inoculated either with PBS or Aβ_wt_. The decreased synaptic density was mainly related to a decrease in the density of postsynaptic markers as seen with Homer (Fig. 5e, h). Unlike Aβ_osa_, Aβ_wt_ did not modulate synaptic densities compared with PBS-inoculated animals (Fig. 5c–h). Synapses in the CA2/3 region and the entorhinal cortex were not affected by Aβ_osa_ or Aβ_wt_ (Fig. 5i-j). Taken together, these results indicate that a single inoculation of Aβ_osa_ has a long-term effect on synapse. Altogether our results suggest that inoculation of Aβ_osa_ increases a cascade of events leading to synaptic impairments, neuronal networks disorganization and cognitive impairments.

### Aβ_osa_ inoculation increases long-term Aβ plaque deposition

**Fig. 6:**
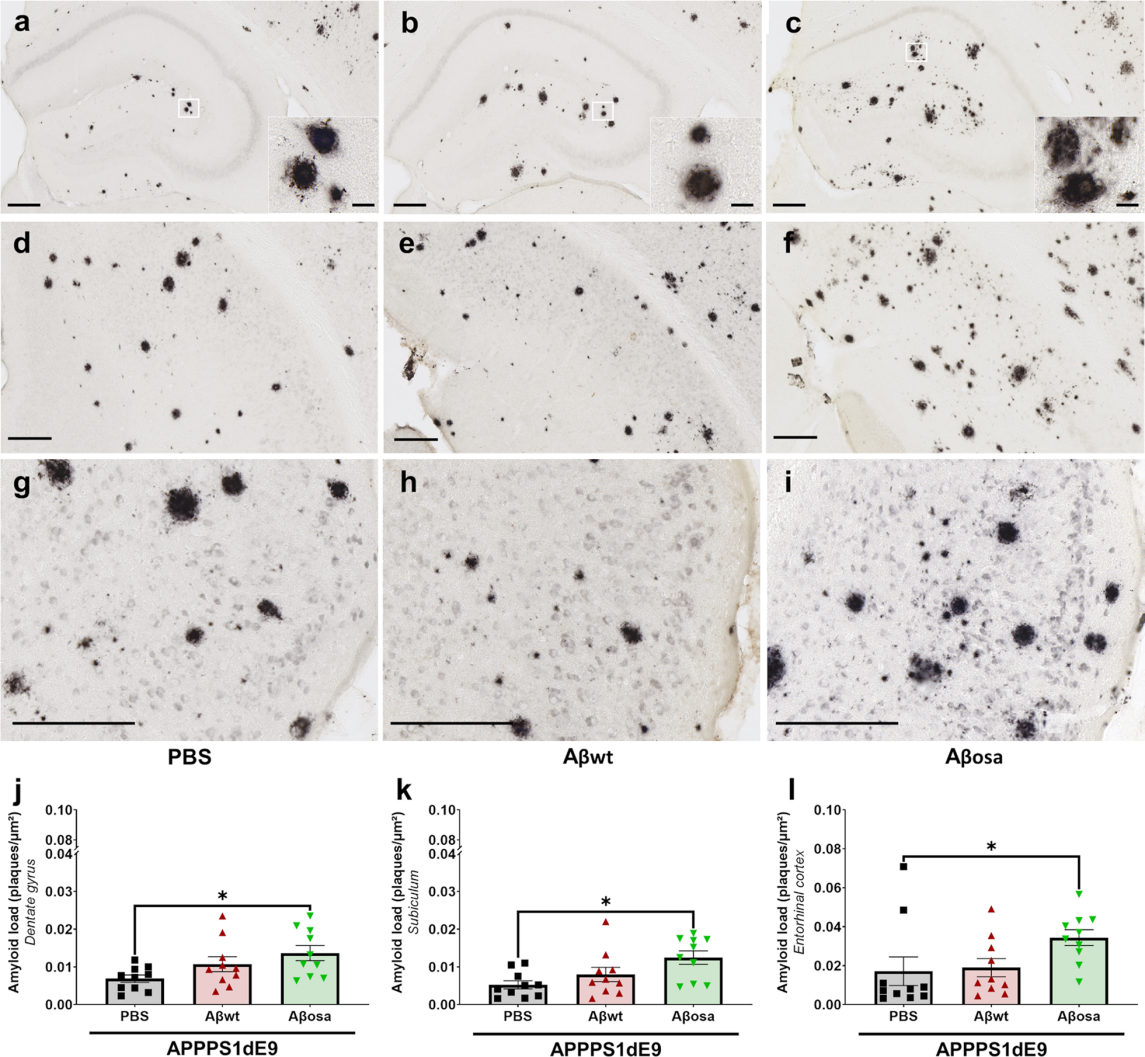
Modulation of Aβ plaque load following inoculation of Aβ variants. Representative images of 4G8 immunolabeling showing Aβ plaque deposition in the dorsal hippocampus (**a-c**), subiculum (**d-f**) and entorhinal cortex (**g-i**) of APP_swe_/PS1_dE9_ mice after PBS, Aβ_wt_ or Aβ_osa_ inoculation in the dentate gyrus. **j-l**. Quantification of amyloid load (4G8-positive amyloid plaques per µm^2^) in the dentate gyrus (**j**), in the subiculum (**k**), and in the entorhinal cortex (**l**). Aβ_osa_ increases Aβ plaque deposition in the dentate gyrus (p = 0.04), in the subiculum (*p* = 0.02), in the entorhinal cortex (p = 0.02). Kruskal–Wallis with Dunn’s multiple comparisons. n_APP/PS1-PBS_ = 10, n_Aβwt_ = 10, n_Aβosa_ = 10 mice. Data are shown as mean ± s.e.m. Scale bars main images: 200 µm; Insets: 20 µm

Four mpi, Aβ plaque load was increased at the inoculation site *i.e.* in the dentate gyrus of mice inoculated with Aβ_osa_ compared to APP_swe_/PS1_dE9_ inoculated with PBS (Fig. 6a–c, 6j). Interestingly, Aβ plaque load was also increased in regions connected to the inoculation site as the subiculum (Fig. 6l) and the entorhinal cortex (Fig. 6d–f, 5g), in animals inoculated with Aβ_osa_. The Aβ load was similar in the CA1 **(not shown)** and CA2/3 **(not shown)** of the three experimental groups. This can be explained by the low amyloid load in these regions. We did not find differences in plaque morphology (Fig. 6a–i). Taken together, these results indicate that inoculation of Aβ_osa_ increases Aβ pathology at 4 mpi.

### Aβ_osa_ inoculation increases Aβ oligomers and modulates APP processing

**Fig. 7:**
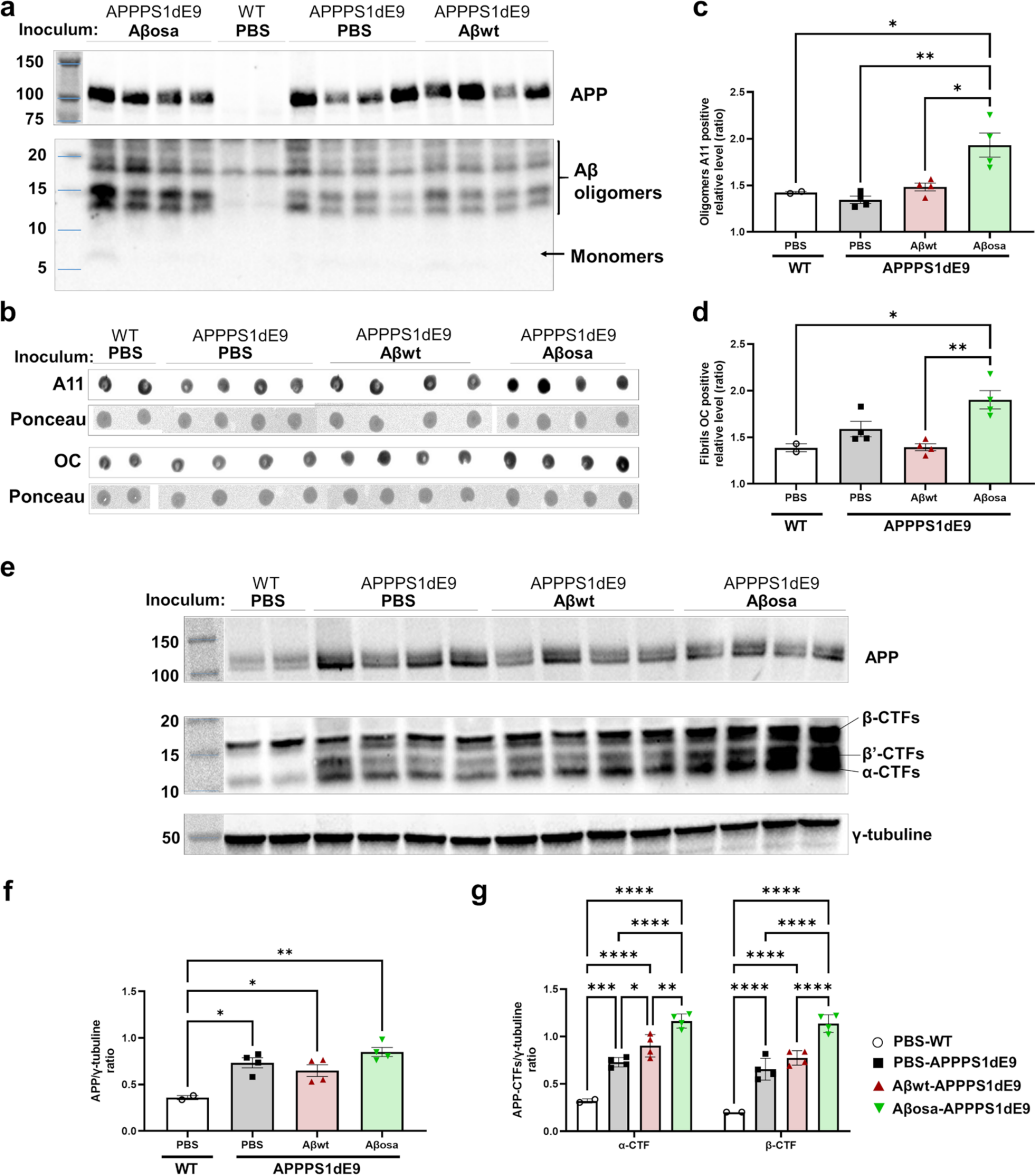
APP proteolysis profiles and oligomerization of amyloid-β peptides at 4 mpi. **a.** Western-blot analysis (6E10 Antibody) of total human APP and Aβ oligomeric species in sarkosyl-soluble extracts of the hippocampus of APP_swe_/PS1_dE9_ mice after PBS, Aβ_wt_ or Aβ_osa_ inoculation at 4mpi. Full length APP runs at an apparent molecular size of 110 kDa, oligomeric forms of Aβ are detected at 15 kDa and 12 kDa. **b.** Dot blot analysis for oligomeric species (A11) and fibrils (OC) in sarkosyl-soluble extract from the hippocampus. **c.** Quantification of relative expression levels of A11 are presented. Aβ_osa_ increased oligomer forms compared to PBS- (p = 0.0019), Aβ_wt_-inoculated (*p* = 0.0106) APP_swe_/PS1_dE9_ and WT mice (*p* = 0.0142). **d.** Quantification of relative expression levels of OC are presented. Aβ_osa_ increased fibrils compared to Aβ_wt_-inoculated (*p* = 0.0038) APP_swe_/PS1_dE9_ and WT mice (*p* = 0.0118) **e.** Western-blot analysis (APP-Cter-17 antibody) of total endogenous APP, APP-CTFs and tubulin in hippocampus lysates (S100K fractions) obtained from wild-type and APP_swe_/PS1_dE9_ mice after PBS, Aβ_wt_ or Aβ_osa_ inoculation. Tubulin staining was used as a marker and loading control. **f-g.** Semi-quantification of APP (APP-Cter-C17 Antibody (**f**)) and of β-CTF/C99 and α-CTF/C83 (APP-Cter-C17 Antibody (**g**)) [39]. Kruskal–Wallis with Dunn’s multiple comparisons. *p < 0.5, **p < 0.05, ***p < 0.005, ****p < 0.0005. nWT_PBS_ = 2, nAPP/PS1_PBS_ = 4, n_Aβwt_ = 4, n_Aβosa_ = 4 mice. Data are shown as mean ± s.e.m

In order to evaluate if oligomer composition is affected by the inoculation of Aβ_osa_ in mice, we fractionated soluble and insoluble Aβ aggregates from the hippocampus by sarkosyl detergent extraction. Western blot analysis of sarkosyl-soluble fraction showed an increase of 15 kDa and 12KDa bands (also referred as 4-mer and 3-mer forms of Aβ oligomers [28]) in Aβ_osa_-inoculated animals (Fig. 7a). On the contrary, Aβ_wt_ did not affect amyloid oligomer profiles as compared to PBS. The enrichment in multimeric/assembled forms of Aβ was confirmed by dot blot analysis using conformational antibodies against oligomers (A11) and fibrils (OC). We observed that both species increased in the hippocampus of Aβ_osa_-inoculated APP_swe_/PS1_dE9_ mice compared to WT mice (Fig. 7b–d). Taken together, these results support the hypothesis that Aβ seeds are able to modulate Aβ aggregation processes up to 4 mpi.

An alternative hypothesis is that Aβ_osa_ modulated APP processing [36]. To test this hypothesis, we assessed APP proteolytic profiles caused by ∝—or β- secretase pathways in the hippocampus at 4 mpi. We quantified by Western blot ∝ -CTF (C83, 9kDA) and β-CTF (C99, 11 kDa) (Fig. 7e–f). We found an increase of ∝ -CTF and β-CTF following Aβ_osa_ inoculation compared to PBS and Aβ_wt_-inoculation in APP_swe_/PS1d_E9_ mice, suggesting that Aβ_osa_ seeds modulate APP processing in vivo (Fig. 7e–f).

## Discussion

We showed that intracerebral inoculation of Aβ_osa_ seeds in the hippocampus of a mouse model of amyloidosis worsens the clinical outcome associated with Aβ deposition 4 mpi. This leads to cognitive and synaptic impairments, reduced functional connectivity between brain regions involved in memory circuits, increased focal Aβ plaque deposition at the inoculation site and spreading of Aβ in the brain as well as increased presence of Aβ oligomers and modulation of APP processing.

### Amyloid deposition and spreading induced by Aβ_osa_

The Aβ deposition induced by Aβ_osa_ inoculation in APP_swe_/PS1_dE9_ transgenic mice can be interpreted using mechanistic models explaining prion diseases. They suggest that in presence of preformed amyloid seeds, newly produced non-β-sheet monomers can change their conformation to assemble into novel aggregated amyloid structures thus inducing a self-propagating process [18]. APP_swe_/PS1_dE9_ transgenic mice express human-like Aβ_1-40_ and Aβ_1-42_ peptides [16] and do not express Aβ_osa_. Thus, likely, Aβ_osa_ increased aggregation of endogenous Aβ_1-40_ or Aβ_1-42_ peptides. Our Thioflavin binding aggregation assay showing that Aβ_osa_ has pro-aggregative properties in presence of Aβ_1-42_ is consistent with this hypothesis. Several studies have shown that inoculum containing Aβ are eliminated from the brain on the days following the inoculation [19, 52]. It is thus reasonable to assume that Aβ_osa_ seeds were eliminated from the brains on the days following their inoculation. The long-term effects of the single inoculation of Aβ_osa_ can thus be explained by an early increase of Aβ deposition induced by Aβ_osa_. As we detected changes of APP processing, another non-exclusive hypothesis is that, following Aβ_osa_ inoculation and subsequent increased Aβ deposition, APP processing was shifted towards increased Aβ production as shown in [36]. This hypothesis however still has to be validated in vivo.

It is interesting to outline that Aβ_osa_ inoculation not only modulated quantitatively the amyloid plaque load, but also increased Aβ oligomers. In addition to a local effect in the dentate gyrus, inoculation of Aβ_osa_ induced increased Aβ deposition in the entorhinal cortex, *i.e.* at distance of the inoculation site. The spreading of Aβ from the inoculum site to interconnected brain regions has already been reported in mice and this has been interpreted as related to a self-propagating process [7, 53]. In our study, it is likely that, the spreading seeds were secondary Aβ seeds produced locally at the inoculation site by the host after Aβ_osa_ inoculation. We can however not rule out that Aβ_osa_ seeds spread in the entorhinal cortex just after its inoculation to induce Aβ aggregation in this region.

### Long-term pathological cascades following Aβ_osa_ inoculation

We found that the cascade of events induced by Aβ_osa_ is able to induce cognitive and synaptic impairments 4 mpi. To our knowledge, long-term effects of synthetic or recombinant Aβ seeds on cognition or synaptic impairments had not been reported. As Aβ is known to induce synaptic deficits either ex vivo or in transgenic mouse models [38, 40, 41], it is reasonable to consider that the increased Aβ plaque deposition and Aβ oligomersinduced by Aβ_osa_ was, at least in part, responsible for the cognitive and synaptic impairments detected in Aβ_osa_-inoculated APP_swe_/PS1_dE9_ mice. Local Aβ plaque deposition may however not be the sole culprit in synaptic impairments as Aβ was not changes in the CA1 after Aβ_osa_-inoculation while synaptic density was reduced. Soluble forms of amyloid could be the culprit. Alternatively, the synaptic impairments could be the consequence of Aβ-related neuronal impairments in projecting neurons. Interestingly, we showed that Aβ-associated pathology induced by Aβ_osa_ occurred locally and was generalized to the entorhinal cortex. We also found that inoculation of Aβ_osa_ affects both local hippocampus functional connectivity and global connections within networks. This contributes to the assumption of an AD-like disconnection syndrome in the inoculated mice. This global disconnection occurs mainly in brain areas connected to the hippocampus and the entorhinal cortex in which Aβ deposits accumulated. Although Aβ build-up seems to trigger the pathological cascade, primary effect linked to Aβ_osa_ it-self cannot be excluded. Notably, the interplay between accumulation of Aβ_osa_ aggregates and synaptic or memory impairments have been hinted by others in several in vitro and ex vivo studies [21, 45–47].

### Differences between a single Aβ_osa_ inoculation in transgenic mice and pathologies developed by patients or mice producing Aβ_osa_

Extracellular Aβ plaques are rare in patients with Aβ_osa_ mutations or in transgenic mice expressing human APP695 with the Osaka mutation (APP_E693Δ_-Tg mice). These mice express the mutation under the mouse prion protein promoter and have a C57BL/6 background [45]. They have an APP expression level similar to those of endogenous mouse APP and they do not show any Aβ plaques even at 24 months but display an age-dependent accumulation of Aβ oligomers within neurons [29, 45]. Our results showing increased Aβ depositions observed in mice following Aβ_osa_ inoculation was unexpected given the lack of Aβ plaques in humans and APP_E693Δ_-Tg mice. Thus, Aβ_osa_ triggers different processes when it is secreted at physiological level and when it is used as an exogenous seed in mice overexpressing Aβ_1-40_ or Aβ_1-42_.

## Limitations of the study

In our study, we chose to study the impact of Aβ_osa_ inoculation in 6-month-old APP_swe_/PS1_dE9_ mice, *i.e.* 4 mpi. In these mice, in the absence of any inoculum, Aβ is continuously secreted and the first Aβ plaques occur in 4-month-old animals, while in 6 month-old animals, they have reached the cortex and the hippocampus. In a previous study, we showed that, in these mice, Aβ plaque load is increased at the inoculation site 4 months after intrahippocampal Alzheimer human brain extract inoculation [24]. Our result with Aβ_osa_ is consistent with this result, but showed a spreading of Aβ plaques and cognitive alterations that were not detected at 4 mpi after human brain extract inoculation. Thus, the pathology induced by Aβ_osa_ seems more severe than that induced by Alzheimer brain extracts. Unexpectedly, at 4 mpi, Aβ_wt_ inoculation had no effect on Aβ plaque deposition. The Aβ_wt_ that we used displayed typical characteristics of Aβ, *i.e.* self-aggregating and synaptotoxic properties in vitro. It is possible that pro-aggregation properties of our recombinant Aβ_wt_ was lower than that of Aβ_osa_ or human Alzheimer extracts. Thus, animal models that show Aβ deposition more slowly are required to dissociate spontaneous and induced deposition induced by Aβ_wt_. Consistently with this suggestion, the induction of Aβ plaques by synthetic Aβ_1-42_ (corresponding to Aβ_wt_) seeds has been reported in mice only after 11 or 13 months post-inoculation in slowly evolving models [43, 44]. A complementary hypothesis is that recombinant Aβ_1-42_ was less toxic than synthetic Aβ_1-42_. To our knowledge, recombinant Aβ_1-42_ seeds have never been used for experimental transmission of Aβ in vivo and it is difficult to extrapolate results from synthetic to recombinant seeds as they display different aggregation properties [9, 50].

## Conclusion

We showed that a single inoculation of Aβ_osa_ induces a long-term cascade of events leading to increased Aβ aggregation and Aβ oligomers in the brain. These events impair synaptic health, leading to cerebral network reorganization and cognitive impairments. This is the first study showing long-term functional toxicity of Aβ seeds. These results suggest that a single, sporadic event as Aβ_osa_ inoculation can worsen the fate of the pathology and clinical outcome several months after the event. Further studies are now required to evaluate links between the structure of Aβ_osa_ and its toxic effects.

## Data Availability

The data that support the findings of this study are available from the corresponding author.

## Abbreviations

Aβ: Amyloid-β
AD: Alzheimer's disease
Mpi: Months post-inoculation
APP: Amyloid precursor protein
PS1: Presenilin 1
WT: Wild-type
ThT: Thioflavin T
SBA: Seed-based analysis
